# Long-Term Follow-Up of Acute Hepatitis B: New Insights in Its Natural History and Implications for Antiviral Treatment

**DOI:** 10.3390/genes9060293

**Published:** 2018-06-12

**Authors:** Stefano Menzo, Claudia Minosse, Donatella Vincenti, Laura Vincenzi, Fabio Iacomi, Paola Zaccaro, Gianpiero D’Offizi, Maria R. Capobianchi

**Affiliations:** 1 Department of Biomedical Sciences and Public Medicine, Università Politecnica delle Marche, Via Tronto 10/a, 60126 Ancona, Italy; 2 Virology Unit, National Institute for Infectious Diseases “Lazzaro Spallanzani”, via Portuense 292, 00149 Rome, Italy; claudia.minosse@inmi.it (C.M.); donatella.vincenti@inmi.it (D.V.); paola.zaccaro@inmi.it (P.Z.); maria.capobianchi@inmi.it (M.R.C.); 3 Infectious Diseases and Hepatology Unit, National Institute for Infectious Diseases “Lazzaro Spallanzani”, via Portuense 292, 00149 Rome, Italy; laura.vincenzi@inmi.it (L.V.); fabio.iacomi@inmi.it (F.I.); gianpiero.doffizi@inmi.it (G.D.)

**Keywords:** HBV, HBsAg, acute hepatitis, genotype

## Abstract

Acute hepatitis B infection (AHB) is still a common viral acute hepatitis worldwide. As vaccination, antiviral treatment, and immigration are bound to affect the epidemiological landscape of HBV infections, and some of its aspects need to be investigated: (1) the circulation of vaccine escape mutants and of primary drug resistant strains; (2) the change in HBV genotype prevalence; and (3) the clinical implications of AHB and the probability of chronification. The serological, virological, and clinical parameters of 75 patients, acutely infected by HBV, were gathered for a retrospective study. Long-term follow up, either to complete seroconversion or for up to five years, was possible for 44 patients. Sequence analysis of the reverse transcriptase/HBsAg and precore regions was performed to investigate the molecular epidemiology and pathogenesis of recent infections by HBV. Genotype distribution in AHB in Italian patients was radically different from that of chronic infections, with a dramatic increase of extra-European genotypes (A1, F), suggesting that a proportion of AHBs are currently related to imported strains. None of the documented infections occurred in vaccinated individuals, while HBsAg variants (potentially vaccine escape variants) were rare and less prevalent than in chronic infections. No drug resistant strains were observed. Spontaneous viral clearance occurred in all but three cases. Time to viral clearance was inversely proportional to liver damage, but HBsAg titer on day 28 and, better still, HBsAg decay from day 0 to day 28 after admission, were the best predictors of chronification. They are, thus, potentially useful to guide antiviral treatment to prevent chronic evolution.

## 1. Introduction

HBV infection is still one of the most impacting viral infections in the world, with a high nosologic burden in most countries, which can be dramatic in those with a high prevalence of infection. Two important factors have profoundly affected its epidemiology worldwide and will affect it further. The first is the implementation of mass vaccination and perinatal infection control schemes. Viruses capable of infecting vaccinated individuals have been identified (HBsAg mutants (for review see [1,2]), but, currently, there are no conclusive data on the impact of escape mutations on the diffusion of HBV in healthy vaccinated populations. The second important factor is the introduction of successful antiviral treatments, which can freeze the disease to inactivity (and stop infectivity). After unsatisfactory results with lamivudine and adefovir, second-generation, high-barrier antivirals, such as entecavir and tenofovir, have overcome the limited potential of the virus to select resistant variants. However, primary resistance to lamivudine, although relatively rare in Europe and in the USA [3,4,5], has been described as worryingly frequent in China [6,7], posing threats to entecavir treatments. In addition, migratory fluxes from countries with a high prevalence of the infection are bound to affect HBV epidemiology in many countries that are currently net recipients of migrants, such as Italy [8]. All the aforementioned factors will certainly, although unpredictably, affect the global trend in the epidemiology of HBV infection, with different effects in different countries. Investigating acute hepatitis B infection (AHB) should provide the necessary data to outline the trends of HBV epidemiology and molecular epidemiology in this rapidly-evolving social and public health context.

AHB currently represents the most frequent form of acute viral hepatitis in Rome, often requiring hospital admission, and may become life threatening in some cases. Symptomatic AHBs rarely become chronic [9,10], however, the few that do will need indefinite treatment. A focused analysis of virological markers in AHB should, therefore, provide knowledge for implementing a rational use of antivirals in this setting. For example, the study of HBsAg kinetics in the early phases of the infection might prove to be a reliable early predictor of chronification, a prognostic marker is currently lacking. In addition, viral genotypic markers, in the context of AHB, might prove useful. Among these, the mutations in the Basic Core Promoter (BCP)/precore region (for review see [1]), need further assessment to clarify: (1) whether precore mutants can be directly responsible for primary infections; and (2) what is the role of different BCP/precore mutations during clearance of the infection in different genotypes and what is their prognostic value. In order to gain a deeper insight into these epidemiologic, pathogenetic, and clinical issues, this study was designed as a retrospective study, investigating a small cohort of AHB patients in a long-term follow-up.

## 2. Materials and Methods

### 2.1. Patients

Seventy-five patients with symptomatic AHB, consecutively admitted and cared for at the Institute for Infectious Diseases ‘Lazzaro Spallanzani’ in Rome, in 2009–2010, were enrolled for the study. Patient characteristics are shown in Table 1.

AHB was defined as an acute illness with a discrete onset accompanied by any of the following signs or symptoms: Fever, headache, malaise, anorexia, nausea, vomiting, diarrhea, and abdominal pain, and either (a) jaundice, or (b) elevated serum alanine aminotransferase (ALT) levels > 100 IU/L. Virological criteria were HBsAg positive and IgM anti-HBc positive (Signal/Cutoff score > 5, to exclude acute-onchronic infections). All patients enrolled in the study were adults and, at the time of admission, signed a written informed consent form stating that their biological samples and data could be stored and used for research purposes. The study was approved by the Ethics Committee of the National Institute for Infectious Diseases ‘L. Spallanzani’ in Rome, Italy with the approval ID n°55 on the 29th October 2008.

### 2.2. Virological Analyses and Viral Load Quantitation

All serologic testing was performed using the Chemiluminescent Microparticle ImmunoAssay (CMIA) technique on the Architect i2000 platform (Abbott Diagnostics, Wiesbaden, Germany). The system provides standardized quantitative results for HBsAg and anti HBsAg, expressed as International Units and milliInternational Units (IU/mL and mIU/mL), respectively (samples exceeding the linear range were quantified after automatic dilution using the instrument for both assays). HBV DNA load was measured using the COBAS TaqMan HBV test (Roche, Pleasnton, CA, USA).

### 2.3. DNA Extraction, Amplification and Sequencing

DNA was extracted from plasma by an automated procedure (Biorobot MDx, Qiagen, Hilden, Germany). All samples were amplified using a proof-reading enzyme (Fast Start High Fidelity, Roche). Direct sequencing was performed on an automated ABI Prism 3130 instrument (Applied Biosystems, Foster City, CA, USA) through the use of Big Dye3.1 cycle sequencing kits, using the same sense and antisense primers. Amplification of the Reverse Transcriptase (RT) region (aminoacid 56 to 357) was performed employing the sense primer HBP1SE: TCTAGACTCGTGGTGGACTTCTC with the antisense primer: ADF2R (as synthesized) TGGGGGTTGCGTCAGCAAACACTTG.

### 2.4. Worldwide HBV Sequence Selection and Phylogenetic Analysis

Sixty-six HBV RT sequences from AHB patients born in Italy (this study) were compared to the corresponding region of isolates with known geographical origins, retrieved from GenBank [11]. A total of 6890 sequences with a geographical origin (from all continents) were downloaded and aligned using ClustalX 2.1 [12]. Incomplete sequences, or those with major indels or other anomalies, were excluded. A first provisional Maximum Likelihood tree was performed. A few sequences, clustering closely in the proximity of the 66 sequences from this study, were selected for further phylogenetic analysis. In addition to these, a minority of the remaining sequences was randomly selected for further analysis (1 every 5 to 1 every 50, depending on the country of origin, in order to obtain a balanced representation). After a second provisional Maximum Likelihood phylogenetic analysis (with a bootstrap test of phylogeny) a few sequences, possibly recombinant strains, generating peripheral isolated clusters, were excluded from the final analysis, leaving a total of 485 sequences, representative of eight genotypes (A to H) from all continents. All phylogenetic analyses were conducted using MEGA version 7 (Kumar, Stecher and Tamura, 2016). The tools in the package were used, both to fit the best evolutionary model relative to the dataset (General Time Reversible), and to perform phylogenetic reconstructions.

### 2.5. Statistical Analysis

When not specifically stated, differences in quantitative parameters were calculated using the Mann-Whitney/Wilcoxon test, as well as differences in frequencies using the chi square test or the Fisher exact test when required. Decay curves were calculated using either the monophasic (HBsAg) or the triphasic (biochemical parameters) decay option in Prism Graph Pad (GraphPad Software v5.01, La Jolla, CA, USA).

## 3. Results

### 3.1. Molecular Epidemiology of Symptomatic Acute Hepatitis B Infection

As a direct comparison between present and past molecular epidemiologies of AHB was not possible, isolates from AHB patients were compared to 424 isolates from Italian Cronic Hepatitis B (CHB) patients, mostly acquired decades ago, collected in the same center, (Table 2).

**Figure 1:**
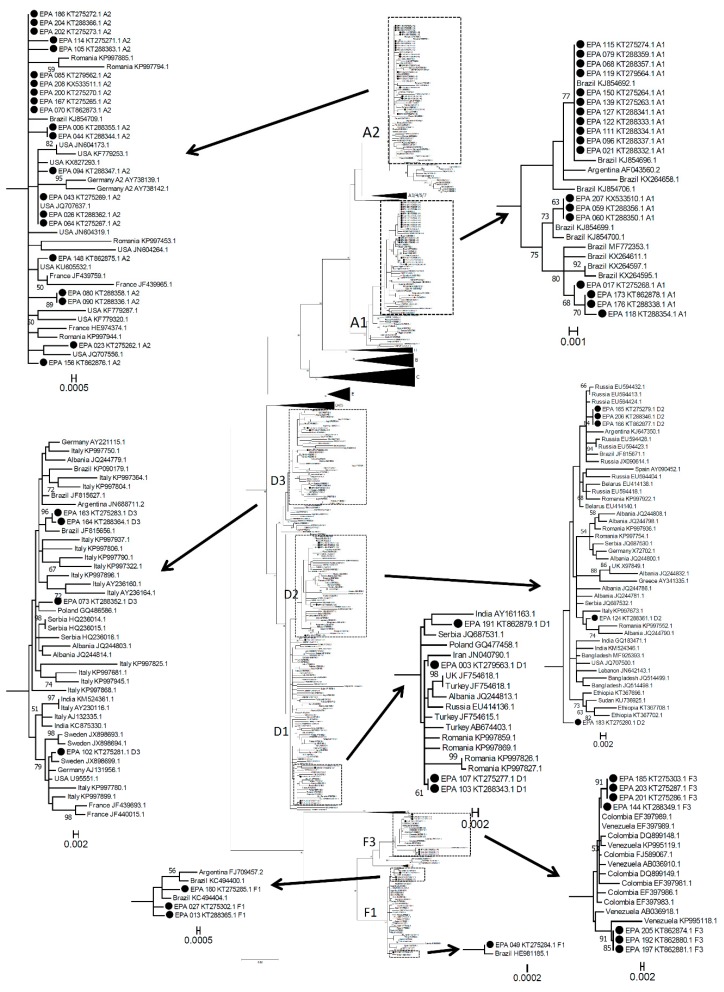
Maximum Likelihood phylogenetic tree of the Reverse Transcriptase (RT) region of HBV including 66 HBV isolates from Italian acute hepatitis B infection (AHB) patients (from this study marked by a dot and indicated as EPA + consecutive identification number + accession number, with the indication of genotype/subgenotype) and 487 isolates from infections worldwide (with geographical origin and accession number). Bootstrap values >50 are displayed. Subtrees representing B, C, G and H genotypes, A3-A7, D4/D5 and F2 subgenotypes (not relevant to the aims of this study) have been collapsed for the sake of space. The boxes in the main tree include subtrees containing the sequences from this study (marked by a black dot). These subtrees have been expanded for a better reading of the sequence names.

Whereas the majority of chronic infections were sustained by the D virus, new infections displayed a greatly increased proportion of A and F viruses, with D3 infections (the most frequent in CHB) reduced to a small minority. In order to investigate the geographical origin of these strains, a phylogenetic analysis was performed, comparing the RT sequence of the isolates from 66 Italian-born AHB patients with those of 485 worldwide isolates (Figure 1), including 40 previous CHB isolates from Italy. In this context, AHB sequences appear in very compact microclusters, mostly unrelated with previous CHB Italian isolates. Genotype A1 strains were closely related to strains from Brazil, A2 strains were related to strains from Eastern Europe, in particular Romania, Russia, and Poland (more than Germany, UK, or the USA), and F from South America (F1 again from Brazil, F3 from Venezuela and Colombia). Even the few genotype D strains were more closely related to those from Eastern Europe (from Romania, Russia, the Balkans) than from Italy. This striking and peculiar epidemiological shift is accompanied by a male gender bias, which is stronger than in CHB (86.4% vs. 74.1%, *p* = 0.0307), suggesting that, not only are imported strains becoming the majority, but also transmission routes leading to HBV infection are changing.

### 3.2. Clinical Outcome, Natural History and HBsAg Clearance

**Figure 2:**
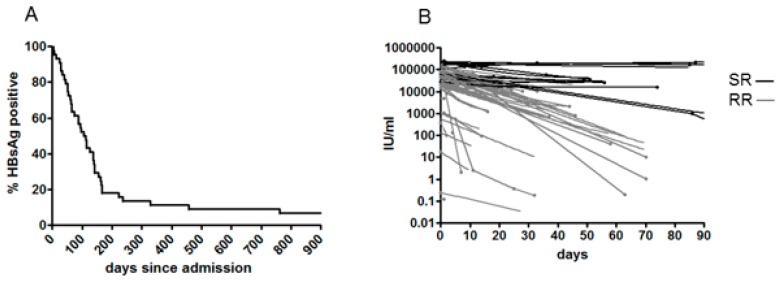
HBsAg clearance in acute symptomatic hepatitis B. Kaplan Meier analysis of HBsAg clearance (**A**) and individual decay curves of HBsAg (**B**) in Rapid Resolvers (RR) (grey) and Slow Resolvers (SR) (black) patients.

All 75 patients were cared for as inpatients for a few days on supportive treatment: Hydration, glucose, and electrolytic balance. Three patients with particularly high levels of liver damage were treated with entecavir until anti-HBsAg seroconversion. After clinical improvement, all patients were discharged, and most were further followed at the same center as outpatients. Clinical data recorded during the hospital stay or collected during follow-up visits were analyzed for the study. None of the patients reported or were documented as having been vaccinated. Twenty-six patients were lost during follow-up after a short hospital stay, 49 were followed as outpatients for a variable amount of time. Of these, 44 were followed to HBsAg clearance or for longer than five years. For the purposes of this study, HBsAg clearance was defined as the intermediate time between the last positive and the first negative result. By this definition, 36/44 patients cleared HBsAg within six months (Rapid Resolvers, RR), as shown in Figure 2A. Of the other eight (Slow Resolvers, SR), three (3/44, 6.8%) developed a true chronic HBeAg+ infection (>4 years now, two of them requiring chronic antiviral treatment). SR were infected with: Four genotype A, two genotype F, and one of genotype D and B respectively. Of these, two genotype A and one genotype B infections became chronic. Genotype D infection in adults showed the lowest probability of lasting longer than six months (6.7% vs. 22% non–D genotypes, not significant).

Quantitative HBsAg values suitable for tracing individual HBsAg decay curves (best fit model: one-phase exponential decay) were available for 37 patients (29 RR and 8 SR). As Figure 2B shows, HBsAg decay is strikingly different in RR and SR patients, even in the first month after admission.

**Figure 3:**
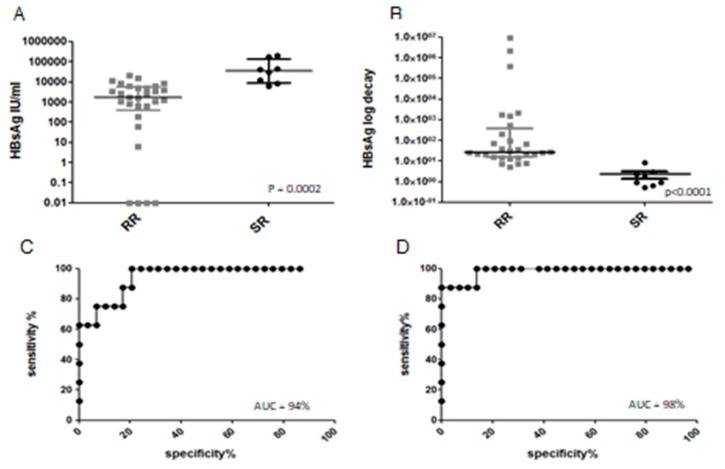
Comparison of HBsAg values and their decay kinetics in RR and SR patients. Absolute HBsAg values on day 28 (HBsAg_d28_) from admission (**A**) and HBsAg decay slopes (expressed as difference in log HBsAg concentration between day 0 and day 28: HBsAg decay_d0–28_) (**B**). Medians with interquartile ranges are shown as horizontal lines. Receive Opeator Corve (ROC) curve for HBsAg_d28_ as predictor of chronification (**C**). ROC curve for log HBsAg decay_d0–28_ as predictor of chronification (**D**). AUC: Area Under the Curve.

Figure 3A shows the differences between RR and SR patients for HBsAg extrapolated at day 28. The differences between RR and SR patients is highly significant, and this parameter can be used as a robust predictive factor for clearing or not clearing HBsAg within six months (ROC curve, Figure 3C, AUC 94%). HBsAg decay slope was also calculated (as the log decrease in HBsAg titer between day 0 and day 28, Figure 3B). The predictive value of HBsAg decrease was even more robust than its absolute determination at day 28 (ROC curve Figure 3D, AUC 98%). When HBsAg clearance was analyzed by genotype, genotypes A and F displayed longer average times to HBsAg clearance (respectively, 241 and 234 days, compared to 101 of genotype D; *p* = 0.068 by the Mann-Whitney test). Quantitative HBV DNA was performed less consistently and in fewer patients, as HBV DNA assessment is not mandatory in AHB guidelines. In 13 patients, it was tested within the first week after admission, ranging from >170,000,000 to 6924, in the other 21 patients it was measured at subsequent follow-up times. Only the eight SR patients showed a high viral load two weeks after admission (>1,000,000 IU/mL), while the 29 RR patients in who blood HBV DNA was measured after two weeks, showed “not detected” or low (<10,000 IU/mL) viral loads (*p* ≤ 0.0001, Mann-Whitney). Despite the data being too scant for a thorough statistical analysis, HBV DNA may also serve as a reliable marker of chronic evolution.

**Figure 4:**
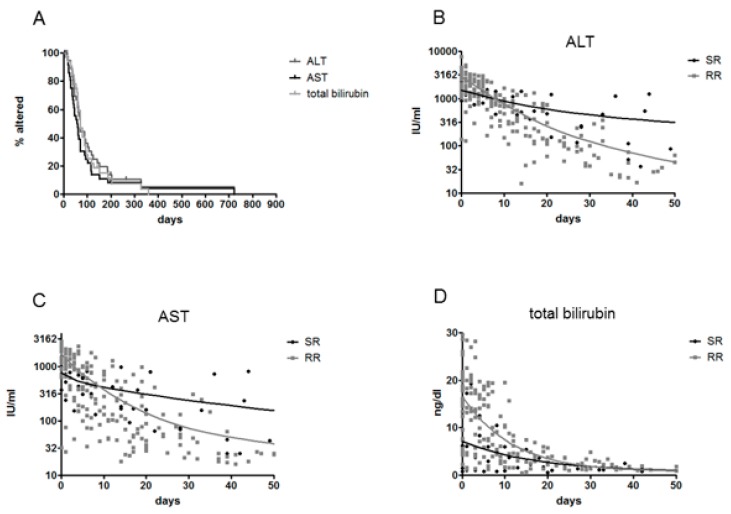
Analysis of the normalization of biochemical parameters. Kaplan Meier analysis of normalization (**A**) of ALT, AST, and total bilirubin in 44 AHB patients with long-term follow-up. Decay of biochemical parameters from the time of peak values. The values of ALT (**B**), AST (**C**), and total bilirubin (**D**) at different time points of patients with follow-up were grouped in scattergrams and the relative best fit-triphasic decay curves were calculated, respectively, for SR and RR.

Both RR and SR patients rapidly stabilized their transaminase and bilirubin values (Figure 4A), but none of the three chronic patients reached the threshold of normality (AST average 64 IU/mL, range 41 to 183). Individual decay curves were not statistically different between RR and SR, and, in contrast to virological parameters, they were not suitable as early predictive markers of chronification. Nonetheless, by pooling together the biochemical data of all patients over time to construct a global three-phase exponential decay model, the decay patterns of biochemical parameters of RR and SR (calculated from the point of peak alteration) were clearly distinguishable, with RR starting with more altered parameters and showing faster normalization, and SR showing lower initial alteration but slower normalization (Figure 4B–D). This suggests that a more pronounced initial parenchymal damage (presumably by the immune response) is associated with a greater chance of clearing infection.

Indeed, in the eight SR patients, peak serum transaminases were significantly lower compared to RR patients (mean ALT 1351 vs. 2626 IU/mL, *p* = 0.001; mean AST 702 vs. 1501 IU/mL, *p* = 0.0033). Similarly, SR showed much lower peak bilirubin (mean 7.6 ng/dL vs. 15 ng/dL, *p* = 0.065). In addition, in 28 patients with an increasing trend in serum bilirubin (hence, closer to the beginning of the infection) the peak alteration of biochemical parameters correlated with the time to HBsAg clearance (AST *R*^2^ = 0.318, *p* = 0.0082, ALT *R*^2^ = 0.145, *p* = 0.0263; total bilirubin *R*^2^ = 0.222, *p* = 0.011, Spearman correlation).

### 3.3. HBeAg/Anti-HBe Seroconversion and Evolution of the Precore Region

The precore genomic region of HBV could be analyzed early after admission in 64 patients. Among the 19 genotype D patients, seven (37%) were HBeAg negative at admission, six of whom (6/19, 32%) with a virus bearing the G1896A mutation, abolishing HBeAg secretion. In patients with G1896A virus, the onset of symptoms, reported at admission, ranged from 3 to 24 days (average 9.5) before the day of sampling for sequence analysis, not statistically different from that (9.9) of HBeAg positive patients with wildtype genotype D virus, suggesting that this mutation either occurs extremely early in the natural history of the infection or is maintained from the previous host of the infecting virus. Follow-up sequences (available for 19 patients) did not reveal any further mutations even in the two wildtype genotype D patients that lost HBeAg in the intervening time. Among the 37 genotype A patients, HBeAg negatives were less frequent soon after admission (*n* = 3, 8%), and none harbored a virus with the G1896A mutations, as expected. One genotype A virus from an HBeAg positive patient displayed a mutation disrupting the precore start codon (mixed base with wild type). One genotype F patient out of eight was HBeAg negative (12.5%), with no mutation. The G1896A mutation was associated to a shorter time to HBsAg clearance in the 44 infections in the whole follow-up cohort (29 vs. 234 days, *p* = 0.0143) and in the 12 genotype D infections (29 vs. 119, *p* = 0.06). Table 3 summarizes the results relative to HBeAg positivity, G1896A precore mutation, and BCP mutations in genotype A and D. No other start, stop or frameshift alterations were detected in the precore region or in the first half of the core gene.

Overall, also BCP mutations seem to affect more genotype D than genotype A virus in AHB. No BCP mutations were found in eight genotype F isolates while the single B and C (HBeAg positive) both harbored the typical (for these genotypes) T1846A variant. Interestingly, BCP/precore mutations were found only in RR patients, however, the difference was not statistically significant.

### 3.4. Mutations in the RT/HBsAg Region

RT/HBsAg sequences were analyzed in 75 AHB patients and no drug resistance mutations were found. As for the HBsAg frame, in only five patients (6.7%) the virus bore mutations in the extracellular loops of HBsAg potentially affecting neutralization in the basal samples: T118V, P127T A128V in one patient (a SR patient), T126I in one patient, A128V in two patients and G145R in one patient. The global frequency of HBsAg variants was lower than in chronic patients (6.7% vs. 11.2%, *p* = 0.311, Fischer exact-test).

## 4. Discussion

In an effort to identify present risk factors for (i) acquiring the infection and (ii) developing CHB, this retrospective study investigated the molecular epidemiology of AHB in Rome and the natural history/outcome of the infection. The genotype distribution among Italian patients appeared very different from that observed in chronic patients from the same center, revealing a consistent increase of formerly less frequent (A2) or rare (A1, F) genotypes from Eastern Europe, South America, and Africa. Hints of this trend had been revealed by previous multicentric studies in Italy [13,14,15], and something similar is happening in Japan [16,17], however, in this peculiar cohort from Rome, the phenomenon appears like an evident subversion of previous molecular epidemiology, possibly because of the more advanced globalization of the capital city. The predominance of genotype A in AHB, in contrast to CHB, cannot not be explained by the lower probability of infections by genotype A virus to evolve to chronicity (previous studies have demonstrated the opposite [13,16,18,19]), but is certainly related to the absence in AHB of the main risk factors for developing CHB: perinatal or childhood transmission. Indeed, the epidemiologic effect of these transmissions has virtually disappeared from the population born in Italy thanks to the prophylactic measures, and we can expect that in the future CHB will derive mostly from evolution to chronicity of adult infections, with a consistent trend towards a shift in genotype distribution also in CHB. The major genotype shift, together with the common notion that sex workers in Italy are mostly from countries in which these ‘new’ genotypes are endemic, suggests that sexual transmission through commercial sex contacts is currently a significant driving force of the HBV epidemics in Italy. A possible other component may be driven by intravenous drug use. In anamnestic interviews on risk factors of this cohort, only 18/72 (25%) declared a sexual risk factor (without further details) and two parenteral drug use (2.8%). Other declared risk factors were: going to the dentist, to the barber, tattooing/piercing, and others. Clearly, self-reported risk factors at anamnestic interviews might be inadequate to quantify the real impact of both sexual and drug-related transmissions. Similar to previous reports from Europe, our study did not show any evidence of vaccine failures or of spread of vaccine escape mutants in the vaccinated population in Italy.

Despite the limitation of its small size, the current study has the advantage of a long follow-up AHB cohort. The outcomes were: One patient suffered from a life-threatening fulminant infection with acute liver failure (1.3% of the entire cohort); 36/44 patients achieved spontaneous viral clearance within six months (81%, among whom the HDV and HCV coinfected patients), eight after six months (18%) and three never cleared HBsAg (‘true’ chronic infections, among whom the HIV positive patient): 6.8% of the follow-up cohort, 3.7% of the entire cohort (as most other patients were certainly lost at follow-up for HBsAg clearance assessed at other centers). A host of studies, mostly in vitro or in animal models, have shown that adaptive T-cell response is critical to obtain viral clearance in acute hepatitis B and acts both by cytotoxic [20,21,22,23] and non-cytotoxic [24,25,26,27,28] mechanisms. In this study, the analysis of the kinetics of virological and hematochemical parameters adds strong in vivo evidence that an aggressive cytotoxic response is indeed required for HBsAg clearance, with the drawback of a more intense (albeit transient) parenchymal damage. Not surprisingly, the only patient who cleared HBsAg within a week of admission is the one who developed a fulminant hepatitis with highest enzymes and acute liver failure (fortunately surviving without transplantation). This patient was a 25-year-old female of Sub-Saharan African origin and was infected with the genotype D3 virus. At admission, she was already HBeAg negative (G1896A) and had only 380 IU/mL HBsAg and 184,023 IU/mL HBV DNA; liver enzymes peaked the day after admission (ALT 2817, AST 7781). The genotype itself affected the clinical outcome of AHB, as previously reported [29]. In our cohort, genotype D patients experienced the highest liver alterations and the lowest HBsAg titers. In addition, all SR patients had non-D genotype infection but one. Together with the fact that genotype D infections tended to show a shorter time to HBsAg clearance, this suggests that infections by D genotype acquired in adulthood have a lower chance of becoming chronic. The role of mutants in the BCP/precore region in determining HBe antigen clearance has been thoroughly studied in different genotypes [30,31,32]. However, its role in HBsAg clearance still needs elucidation. This study demonstrates for the first time the association between the G1896A mutation and a shorter time to HBsAg clearance, extending the observation that it can occur very early after infection (or could indeed be transmitted, as previously suggested [33]). Although in chronic infection G1896A has been controversially suspected of being related to decompensated cirrhosis or cancer [34,35], in acute hepatitis it could be rather considered as a marker of a potent cytotoxic immune response (at genotype parity), generating more severe liver alterations [36] but most successful at clearing the infection. This apparent discrepancy could be reconciled in a model where G1896A (independently of its molecular effects) is selected as an ‘escape’ mutation from T cell response: It exists as a minor variant (as recently demonstrated [37,38,39]) and emerges only after the bulk of the hepatocytes infected by the WT virus has been cleared (sometimes violently as in fulminant AHB) and viremia has been reduced by a few logs. As a consequence, it appears very early in the presence of a strong response (acute hepatitis), while it takes much longer when the response is insufficient (in chronic hepatitis) and is therefore found more frequently in older chronic infections than in recent chronic infection. Possibly, its association with decompensated cirrhosis or cancer is a simple chronological association.

An interesting finding emerging from the long follow-up of this cohort is that not all infections lasting more than six months do necessarily become ‘chronic’: more than half of SR patients spontaneously cleared HBsAg in the subsequent months and within two years. Nonetheless, it would be very important to identify patients with difficulties in clearing the infection, in order to intervene with some form of treatment, as others have suggested and attempted. Antiviral treatment with nucleoside inhibitors has been applied with the main objective of reducing parenchymal damage [40,41], but this approach resulted in prolonged rather than shortened time to HBsAg clearance. By contrast, anecdotic evidence suggests some efficacy of interferon treatment [42], despite controlled trials on the efficacy of treatment in AHB in reducing chronification showing that indiscriminate treatment does not yield a significant effect compared to controls [43,44]. However, these studies involved very small populations of patients, nearly all of whom resolved also in the control group characterized by short follow-up time, with insufficient power to detect any effect. The question whether treatment of AHB may be effective at avoiding evolution to chronicity is still open.

In this study cohort, the number and frequency of quantitative HBsAg determinations allowed us to investigate for the first time this parameter as an early outcome predictor. The results indicate that both HBsAg at day 28 and (better still) the kinetics of HBsAg in the first four weeks from admission are optimal predictors of the chance of clearing HBsAg within six months. An HBsAg decay of less than 0.6 log in HBsAg titer at day 28 should therefore be considered for initiating early treatment only in patients who might really benefit from it. At this cut-off the sensitivity for detecting slow resolvers would be 87.5%, and specificity 100%, while with 1 (log of decay) as a cut-off, the sensitivity would be 100% and the specificity 86.2% (in this cohort). HBV DNA had been previously suggested for the same purpose [45], and this study confirms its potential, however, the advantages of HBsAg monitoring over DNA are evident in terms of simplicity and cost. Dedicated clinical trials should be designed to establish the efficacy of such a strategy.

## Figures and Tables

**Table 1:** Mean and standard deviation of age, anagraphical, biochemical parameters, and virological parameters in 73 acute hepatitis B infection (AHB) patients grouped by the most frequent genotypes (A, D or F). Two patients with genotype B and C virus were not included.

Genotype	A	D	F
Age (years)	42.8 ± 13.1	41.2 ± 1.5	40.9 ± 10.5
Gender (f/m)	8/36	4/16	2/9
AST, IU/mL	2358 ± 684 ^a^	3234 ± 677 ^b^	1264 ± 885
ALT, IU/mL	1491 ± 914 ^c^^,^^d^	1865 ± 1773 ^e^	757 ± 916
Total bilirubin, ng/dl	14.8 ± 8.7 ^f^	17.6 ± 8.9 ^g^	10.3 ± 11.4
HBsAg, logIU/mL	4.64 ± 0.57 ^h^	3.66 ± 1.70	4.28 ± 1.12
HBeAg (−/+ at admission)	4/40	7/13	1/10
HIV (−/+ at admission)	43/1	20/0	11/0
HCV RNA (−/+ at admission)	43/1	20/0	11/0
HDV (−/+ at admission)	43/1	20/0	11/0

Alanine aminotransferase (AST), aspartate aminotransferase, (ALT), total bilirubin and HBsAg values used for this table were the highest measured at any time for each patient. ^a^ Difference vs. genotype F: *p* = 0.0026; ^b^ Difference vs. genotype F: *p* = 0.0005; ^c^ Difference vs. genotype D: *p* = 0.0388; ^d^ Difference vs. genotype F: *p* = 0.0043; ^e^ Difference vs. genotype F: *p* = 0.0016; ^f^ Difference vs. genotype F: *p* = 0.0378; ^g^ Difference vs. genotype F: *p* = 0.0071; ^h^ Difference vs. genotype D: *p* = 0.0373.

**Table 2:** Differences in genotype/subgenotype prevalence in AHB and CHB patients from the same clinical center in Rome.

	AHB	CHB	*p*
A1	19 (28.8%)	23 (6%)	<0.0001
A2	21 (31.8%)	59 (14%)	0.0005
B	1 (1.7%)	0 (0%)	ns
C	1 (1.7%)	0 (0%)	ns
D1	5 (7.7%)	50 (13%)	ns
D2	4 (6.1%)	45 (11%)	ns
D3	4 (6.1%)	227 (52%)	<0.0001
D4	0 (0%)	4 (1%)	ns
F	11 (16.7%)	7 (2%)	<0.0001
G	0 (0%)	4 (1%)	ns
H	0 (0%)	1 (0%)	ns

ns: not significant.

**Table 3:** Prevalence of HBeAg positivity, incidence of chronification and prevalence of BCP/precore mutations in genotype AHB patients infected with genotype A or D HBV.

	A, *n* = 37	D, *n* = 19	*p* (Fisher)
HBeAg—at admission	3 (8%)	7 (37%)	0.01
chronic infection	2 (6%)	0	>0.1
G1896A	0	6 (32%)	0.0006
C1653T	0	1 (5%)	>0.1
T1753A	1 (3%)	1 (5%)	>0.1
A1762T	0	1 (5%)	>0.1
G1764A	1 (3%)	3 (16%)	0.09
T1846A	0	2 (11%)	>0.1
G1899A	2 (6%)	1 (5%)	>0.1
any mutation	4 (11%)	8 (42%)	0.0075
